# Evidence that the Aso-3 caldera-forming eruption (southwest Japan) marks the termination of Marine Isotope Stage (MIS) 6

**DOI:** 10.1016/j.quascirev.2026.109837

**Published:** 2026-04-01

**Authors:** D. McLean, P.G. Albert, G. Jones, R.A. Staff, A. Francke, S.O. Vineberg, J.J. Tyler, M. Saito-Kato, T. Sagawa, K. Kaneko, H. Buckland, T. Suzuki, J.-I. Kimura, Q. Chang, H. Hoshizumi, Y. Miyabuchi, C.J. Manning, K. Yamada, I. Kitaba, K. Ikehara, SG14 Project Members, T. Nakagawa, V.C. Smith

**Affiliations:** a Research Laboratory for Archaeology and the History of Art, University of Oxford, Oxford, OX1 3TG, UK; b Department of Geography, Swansea University, Swansea, Cymru, SA2 8PP, UK; c Discipline of Earth Sciences, School of Physics, Chemistry, and Earth Sciences, University of Adelaide, 5005, Adelaide, Australia; d Department of Geology and Paleontology, National Museum of Nature and Science, 110-8718, Tokyo, Japan; e Institute of Science and Engineering, Kanazawa University, Japan; f Graduate School of Human and Environmental Studies, Kyoto University, Sakyo-ku, Kyoto, 606-8501, Japan; g Department of Geography, Tokyo Metropolitan University, Tokyo, 192-0397, Japan; h Volcano and Earth's Interior Research Center, Institute for Marine Geodynamics, Japan Agency for Marine-Earth Science and Technology (JAMSTEC), Yokosuka, Kanagawa, 237-0016, Japan; i AIST, Geological Survey of Japan, Japan; j Centre for Water Cycle, Marine Environment and Disaster Management, Kumamoto University, Kurokami Chuo-ku, Kumamoto, 860-8555, Japan; k Department of Earth Science, Royal Holloway University of London, UK; l Research Centre for Palaeoclimatology, Ritsumeikan University, Shiga, 525-8577, Japan; m Research Institute of Geology and Geoinformation, Geological Survey of Japan, AIST, Tsukuba, Japan; n Fukui Prefectural Varve Museum, Wakasa, 919-1331, Japan

**Keywords:** Aso-3, Tephrochronology, Lake Suigetsu, Glass shard geochemistry, Termination II

## Abstract

The Aso-3 caldera-forming event of Aso volcano was one of the largest eruptions of the Quaternary period, blanketing vast regions of Japan and surrounding seas in ash. However, uncertainties surrounding the eruption age and geochemical variability have limited its utility as a robust time-stratigraphic marker. Distal occurrences previously attributed to Aso-3 span a broad temporal window (135–110 ka) and glass shards often lack compositional agreement with those of proximal datasets. Here, we re-evaluate the characteristics of Aso-3 using new stratigraphic and geochemical evidence from proximal and distal settings. In the Lake Suigetsu sediments, three Aso tephra layers are newly identified, including a 3 cm thick layer at ∼133 ka with glass shards that compositionally span the proximal Aso-3 range. Additionally, we identify a compositionally identical Aso-3 cryptotephra in the Sea of Japan (core U1427A). Combined stratigraphic, geochemical, and palaeoenvironmental evidence (pollen, diatom and benthic δ^18^O) from these records confirms that Aso-3 occurred prior to Marine Isotope Stage (MIS) 5e, during the termination of MIS 6. This establishes Aso-3 as a regional isochron, aiding synchronisation of paleoclimate records across the glacial–interglacial transition (Termination II). Our findings caution against correlating to Aso-3 based on partial geochemical matches, given that Aso experienced numerous explosive eruptions responsible for widespread ash dispersals throughout MIS 6 and 5.

## Highlights

• The stratigraphic position of the large and widely-dispersed Aso-3 eruption is constrained.
• Climate proxies indicate that the eruption occurred at the MIS 6–5e transition.
• Aso-3 is a valuable isochron for synchronising Termination II records in East Asia.
• Glass geochemistry and age control are critical for identifying the Aso-3 tephra.
• Other Aso-derived MIS 6 tephras are also identified in Lake Suigetsu and marine core U1427A.

## 1 Introduction

Volcanic ash (tephra) layers are extensively preserved in lakes, soils, and marine sediments across East Asia, recording some of the most explosive eruptions of the Quaternary. These deposits not only testify to the frequency and magnitude of past events (e.g., Crosweller et al., 2012; Jenkins et al., 2018; Uesawa et al., 2022) but serve as powerful chronological markers for synchronising and dating sedimentary archives. Japan, one of the world's most volcanically active regions, has produced numerous widespread tephra markers (Machida and Arai, 2003). Many of these layers form the backbone of regional chronostratigraphic frameworks, which align records of climate change, human activity, and environmental response (Machida and Arai, 1983; Suzuki et al., 2011; Moriwaki et al., 2011, 2016; Smith et al., 2013; Ikehara, 2015; Chen et al., 2019; Albert et al., 2019; Tam and Yokoyama, 2021). Widespread and well-characterised tephra can synchronise sequences across thousands of kilometres, offering a rare opportunity to resolve temporal relationships between disparate archives in the East Asian/Pacific region and those beyond (e.g., Bourne et al., 2016; Sun et al., 2014; Ikehara et al., 2006; McLean et al., 2016; Oppenheimer et al., 2017; Costa et al., 2024; Davies et al., 2024).

While the Late Pleistocene and Holocene tephrostratigraphic framework in Japan is well established, securely dated marker layers beyond 100 ka remain limited. This is especially true for the transition from Marine Isotope Stage (MIS) 6 to 5e (∼135–116 ka; Lisiecki and Raymo, 2005), a period of global climate reorganisation known as Termination II. This interval, marking the end of the penultimate glaciation and onset of the last interglacial, was characterised by rapid warming, ice sheet retreat, sea-level rise, and major reorganisation of ocean–atmosphere systems (Hearty et al., 2007; rant et al., 2012). In Japan, it also saw intensification of the East Asian Summer Monsoon (EASM) and spatially complex environmental responses (Sagawa et al., 2018; Tam and Yokoyama, 2021). Despite growing interest in this interval for understanding the rates, sequencing, and feedbacks of large-scale climate transitions relevant to future change, high-resolution synchronisation of Termination II records across East Asia remains challenging due to a scarcity of a robust chronologies for MIS 6 and 5e sedimentary archives (e.g., Tam and Yokoyama, 2021; Francke et al., 2025).

**Fig. 1:**
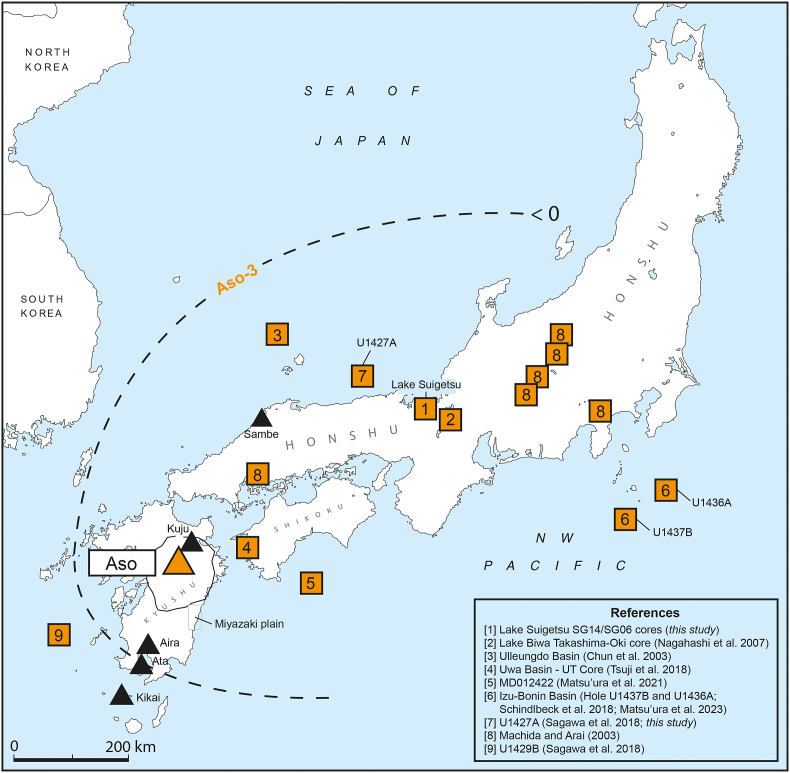
Location of Aso caldera in central Kyushu (orange triangle) along with other volcanoes that are mentioned within the text (black triangles). Key sedimentary records (terrestrial, marine, and lake) that have ash deposits attributed to the Aso-3 caldera-forming eruption are shown as orange squares. The dashed line indicates the Aso-3 dispersal isopach in cm, as defined by Machida and Arai (1994), and marks the known limit of visible ash. (For interpretation of the references to colour in this figure legend, the reader is referred to the Web version of this article.)

The Aso-3 tephra, erupted during a Magnitude (M; Pyle, 2000) 7.2 caldera-forming eruption at Aso volcano in southwestern Japan (Fig. 1), offers significant potential to improve these chronological issues. This enormous eruption deposited over 180 km^3^ of tephra across the Japanese archipelago and surrounding marine basins (Machida et al., 1985; Ono et al., 1977; Machida and Arai, 2003; Kaneko et al., 2015; Kawaguchi et al., 2021). However, its age and stratigraphic position is currently uncertain. Distal tephra layers tentatively linked to Aso-3 have been reported from terrestrial, lacustrine, and marine archives (e.g., Chun et al., 2004; Nagahashi et al., 2004; Schindlbeck et al., 2018), but their reported ages span ∼135 to ∼110 ka, and their geochemical glass signatures are inconsistent. Although the glass compositions are distinctly Aso in composition (i.e., >3 wt % K_2_O; Albert et al., 2019; McLean et al., 2020a), some layers are compositionally homogeneous and rhyolitic, whilst others span broader ranges, including trachy-andesitic and basaltic shards. In some cases, multiple glass-rich horizons are separated by tens of millennia, suggesting reworking, eruption heterogeneity, or misattribution. Compounding this, recent proximal investigations show substantial explosive activity occurred between caldera-forming events (Hoshizumi et al., 2022; Vineberg et al., 2025), raising the possibility of miscorrelation of these Aso distal layers.

The focus of this study is to resolve the age and climato-stratigraphic position of the Aso-3 tephra and define the characteristics that are essential for using this time-stratigraphic marker. We present new glass geochemical data from proximal deposits of Aso volcano, covering eruptive activity throughout MIS 6, and integrate these with stratigraphic and chronological evidence from two key distal sedimentary archives. These include Lake Suigetsu (SG06/SG14 core; central Honshu), arguably one of the most detailed tephra records in Japan, and IODP marine core U1427A (Sea of Japan) (Fig. 1).

### 1.1 Aso and its eruptive history during MIS 6–5

Aso caldera (32°53′04’’N, 131°06′14’’E), located in central Kyushu (Fig. 1), is the most active volcanic centre in the southwest Japan arc. The Aso caldera is large, measuring approximately 18 × 25 km, and has been shaped by at least four major caldera-forming eruptions with magnitudes between M7 and M8 (Ono et al., 1977; Watanabe, 1978; Miyabuchi, 2009). The Aso-1 (∼266 ± 14 ka) and Aso-2 (141 ± 5 ka) eruptions each produced bulk volumes exceeding 50 km^3^ (Ono et al., 1977; Matsumoto et al., 1991; Kaneko et al., 2015). Aso-3 (∼135–115 ka), the penultimate caldera-forming eruption, is estimated to have erupted more than 180 km^3^ of material (M > 7.2; Hoshizumi et al., 2024). The most recent and voluminous event, Aso-4 (∼86–87 ka), produced an estimated total volume exceeding 900 km^3^, based on combined proximal pyroclastic density current (PDC) and distal tephra-fall deposits, indicating a magnitude of at least M8.0 (Takarada and Hoshizumi, 2020).

The Aso-2 eruption is the oldest caldera-forming event at Aso known to have occurred in MIS 6, and the ash has been identified in two key distal records to date, Lake Biwa (unit BT43; Nagahashi et al., 2004) and the NW Pacific marine core MD012422 (unit G18; Matsu'ura et al., 2021). Proximal exposures of the complete eruptive succession of Aso-2 are limited, with published glass compositions reported only for the terminal phase (i.e., Aso-2T; Matsu'ura et al., 2021). This is the main deposit typically found unwelded and with glass suitable for analysis. Between the caldera-forming events of Aso-2 and 3, other significant Plinian events were also frequent. Outcrops of these have been identified in proximal sections that are only exposed east of the caldera (e.g., Aso-R, -OPQ and -U; Ono et al., 1977; Ono and Watanabe, 1983), but had not been geochemically characterised in detail prior to this study (see Section 2.1.).

The enormous Aso-3 caldera-forming event generated a Plinian column (unit 3W) and widespread pyroclastic density currents (PDCs; units 3A, 3B and 3C) (Ono et al., 1977; Watanabe and Ono, 1969; Watanabe and Fujimoto, 1991; Kamata, 1997; Kaneko et al., 2015; Takarada and Hoshizumi, 2020). The ignimbrite alone covers ∼2500 km^2^ around the caldera, extending up to 50 km west and to the coastline and 70 km east of the caldera (Kaneko et al., 2015). Geochemical and isotopic (Sr, Nd, Pb) analyses of the eruption deposits suggest the eruption tapped a stratified magma chamber comprising silicic, intermediate, and mafic layers (Kaneko et al., 2007, 2015; Keller et al., 2023). Major element glass compositions of these proximal units are presented by Kaneko et al. (2015) and are supplemented (additional major and trace element datasets) as part of this study.

Explosive activity continued between Aso-3 and Aso-4 (∼86–87 ka), with at least 37 explosive eruptions and associated tephra units recorded in proximal successions (Hoshizumi et al., 2022; Vineberg et al., 2025). These include scoria, pumice lapilli, and ash fall deposits, grouped into five eruptive stages based on stratigraphy, paleosol development, and the physical and compositional characteristics. Three of these eruptions (Aso-ABCD, Aso-EF and Aso-HI) were widely dispersed and are preserved as visible/cryptotephra layers in the Lake Suigetsu (SG14) sequence 525 km from Aso (summarised in Vineberg et al., 2025). The distal records also reveal more frequent activity at Aso in the 10 kyrs leading up to the Aso-4 caldera-forming eruption. These correlations to the well-dated Lake Suigetsu core help constrain the eruption age estimates and indicate that in some cases short time-periods elapsed between widespread ash fall events.

### 1.2 Aso-3 distal tephra

**Fig. 2:**
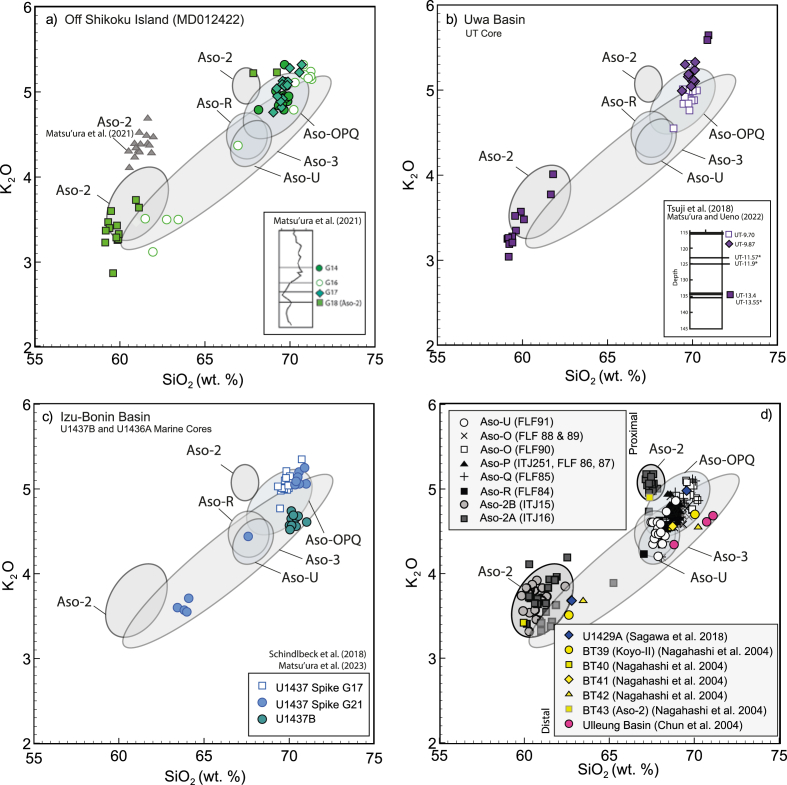
Available published glass compositions (SiO_2_ vs K_2_O) of the distal ash layers that have been attributed to the Aso-3 tephra in (a) marine core MD012422 (∼270 km from Aso; Matsu'ura et al., 2021), (b) Uwa basin UT core (∼150 km from Aso; Tsuji et al., 2018; Matsu’ura et al., 2021) and (c) cores from the Izu-Bonin arc (∼800 km from Aso; Schindlbeck et al., 2018; Matsu'ura et al., 2023). (d) Shows the reported average glass shard compositions for tephra layers reported in the Sea of Japan Core U1429A (∼650 km from Aso), Lake Biwa (BT units; ∼570 km from Aso; Nagahashi et al., 2004) and the Ulleung Basin (Chun et al., 2004). The grey fields are the compositional range of the proximal units (Aso-3 = combined 3W, 3A, 3B, and 3C; Aso-2 = combined Aso-2B -2A; *this study*), with the individual analyses for the intra-caldera events (Aso-U, Aso-OPQ -R; *this study*) and Aso-2 (*this study)* shown in (d).

Distal tephra layers attributed to the Aso-3 eruption have been identified across a range of depositional environments in Japan (Fig. 1). These include terrestrial (e.g., Matsumoto et al., 1991; Machida and Arai, 1994), coastal (e.g., Machida et al., 1991; Shimoyama et al., 1999), lacustrine (e.g., Nagahashi et al., 2004), and marine settings (e.g., Machida and Arai, 1992; Chun et al., 2004; Schindlbeck et al., 2018; Tsuji et al., 2018; Matsu'ura et al., 2021, 2023; Matsu'ura and Ueno, 2022). Together, these widely dispersed occurrences are not stratigraphically consistent, and there are differences in the glass compositions, particularly in the compositional range. This implies that they may not all be associated with the same Aso eruption and thus, the unique characteristics of the Aso-3 tephra need to be identified to facilitate robust correlations. The available glass compositions for these reported layers are shown in Fig. 2.

Machida and Arai (1992, 1994) were among the first to report Aso-3 fall deposits in distal settings across Honshu and the Sea of Japan (Fig. 1). They noted that the fine ashes were characterised by bubble-walled glass shards with trachy-dacitic compositions (65–71 wt% SiO_2_ and >4 wt% K_2_O) (Machida and Arai, 2003). Aso-3 tephra was also reported in coastal terrace deposits, such as the Miyazaki Plain, at elevations up to 21 m a.s.l. (marked on Fig. 1). Machida and Arai (1994) found these layers stratigraphically beneath key marker tephras including the Toya, Ata, Ontake Pumice 1 (On-Pm1), and the Sanbe Kusuki (SK), which constrained the age to >100 ka. Furthermore, direct radiometric ages of proximal Aso-3 pyroclastic flow deposits using K–Ar, fission-track, and thermoluminescence methods yielded ages between ∼103 and 123 ka (Okaguchi, 1978; Matsumoto et al., 1991), placing the eruption prior to 123 ka and within MIS 5d.

Five visible Aso-derived tephras (BT39–BT43 and Ky-I) have also been identified within MIS 6 and MIS 5e sediments at Lake Biwa (Takashima-oki core, ∼570 km from Aso), where marine isotope stages are defined by correlation of the lacustrine oxygen-isotope stratigraphy to the global MIS framework and supported by widespread tephra correlations (Nagahashi et al., 2004). The average glass shard compositions for each of the units is shown in Fig. 2d. BT43 was correlated as the first distal fall deposit of Aso-2, and contains bimodal glass populations (59–61 wt% and 67–68 wt% SiO_2_). It is positioned within mid-MIS 6 sediments, and the depositional age was estimated as ∼140–145 ka (Nagahashi et al., 2004). Layer Ky-I (∼5 cm thick) was attributed to the main distal fallout layer of Aso-3. Its glass compositions show a wide range, including a lower SiO_2_ (∼62.62 ± 0.76 wt%; 1σ) and a higher SiO_2_ population (∼67.37 ± 0.38 wt%). This Ky-I layer is located within MIS 6, prior to the interglacial MIS 5e. The units stratigraphically above, BT40 and BT41 (both 0.5 cm in thickness), are compositionally homogeneous (SiO_2_ ∼68.7 wt%), and suggested as later phases of Aso-3.

Chun et al. (2004) identified an Aso-derived tephra in several cores extracted across the Ulleung Basin (Sea of Japan) which ranged in thickness from 1 to 3 cm (cores 95PC-6, 95PC-9, 95 PC-10). The layer is stratigraphically positioned at the MIS 6–5e transition (∼133 ka) in all cores, and comprised of pumice lapilli with rhyolitic glass compositions, though only limited geochemical data (i.e., three analyses) were presented (Fig. 2d). Further south, Sagawa et al. (2018) reported a compositionally heterogeneous Aso-3 tephra layer (24 cm thick) in marine core U1429A (East China Sea; ∼650 km from Aso) at a depth of 69.8 m. Similarly to the Ulleung Basin, the associated benthic δ^18^O profile placed the eruption just prior to Termination II.

More complex tephrostratigraphy has been described from the Uwa Basin (UT core), located closer to Aso on Shikoku Island (i.e., ∼150 km east; Fig. 1). Initially documented by Tsuji et al. (2018) and revised by Matsu'ura and Ueno (2022), this sequence contains multiple Aso-derived ash-rich layers separated by sediment: three attributed to Aso-3, and four to Aso-2 (Fig. 2). The UT-9.87 unit, tentatively correlated with Aso-3, is dated to ∼112.7 ka based the core age-model and lies within MIS 5c–5e strata. This layer contains exclusively rhyolitic glass shards. Older units such as UT-14.55 were linked to a late-stage Aso-2 subunit (Aso-2T), dated to ∼141 ka, though compositionally similar layers appear above and below (e.g., UT-13.40, -13.55, -14.71, -19.53, -27.21), suggesting other explosive eruptions are recorded and/or post-depositional reworking of some tephra.

Further Aso-derived layers were identified by Matsu'ura et al. (2021) in marine core MD012422, located off Shikoku Island (∼100 km from Aso). Glass shard concentration “spikes” were used to locate the tephra horizons. Three spikes (G14, G16, and G17) were attributed to Aso-3 activity and labelled Aso-3iii (∼131 ka), Aso-3ii (∼134 ka), and Aso-3i (∼137 ka), respectively, based on the age–depth model of Ikehara et al. (2006), which integrates planktonic foraminiferal δ^18^O stratigraphy, dated widespread tephras, calcareous nannofossil biohorizons, and radiocarbon ages in the upper core. G14 and G17 contain unimodal glass populations with SiO_2_ >68 wt% and K_2_O >4.8 wt%, while G16 shows bimodal SiO_2_ distribution (Fig. 2). Chronologically, G16 aligns with the MIS 6–5e boundary, while G14 is associated with the MIS 6 glacial maximum (140–133 ka; Ikehara et al., 2006). An older peak (G18) in the sequence has been correlated to Aso-2 based on its compositional overlap with BT43 in Lake Biwa (Matsu'ura et al., 2021), although the proximal unit analysed of Aso-2T did not span its full range (Fig. 2a).

## 2 Study sites

To investigate the eruptive activity of Aso volcano across MIS 6–5e, and to assess the geochemical composition of the Aso-3 tephra, we utilised sedimentary records in both the proximal and distal realms.

### 2.1 Proximal sites

Proximal exposures of the volcanic sequence were predominately investigated east of Aso caldera, where outcrops are well-preserved and accessible.

**Fig. 3:**
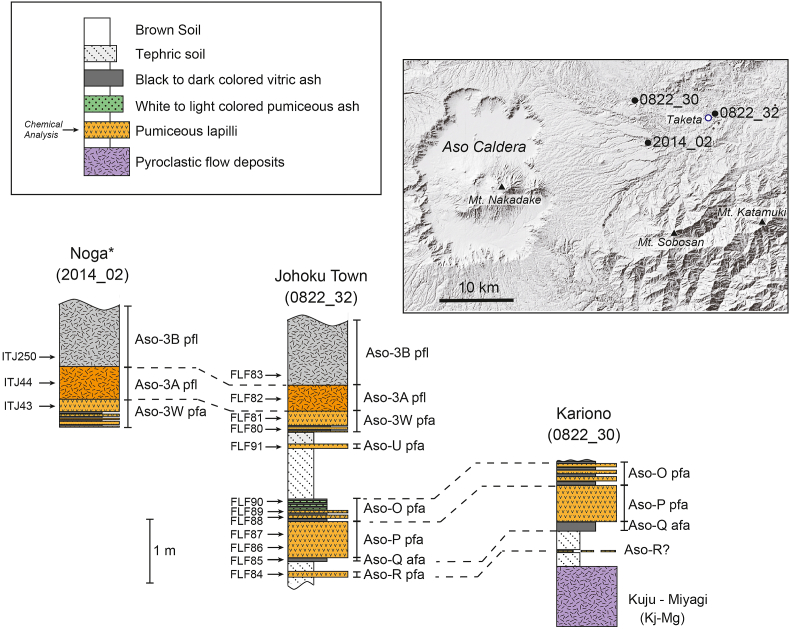
Stratigraphic correlation of key proximal tephra sequences logged at sites east of Aso caldera. The exposure at Johoku Town contains three phases of the Aso-3 caldera-forming event (Kaneko et al., 2015; this study), which are also identified at Noga (Albert et al., 2019). Pre-Aso-3 units are also well-preserved at Johoku Town and include Aso-R, Aso-OPQ and Aso-U. The glass compositions for these units have been analysed as part of this study. Pyroclastic flow deposits of the Kuju-Miyagi (Kj-Mg) deposit (Kuju volcano; Fig. 1) are identified stratigraphically below a soil unit above Aso-R at the site Kariono. See the manuscript text for further details.

The Aso-3 succession (units 3W, 3A, 3B, and 3C) was previously described by Kaneko et al. (2015), who established the major element geochemical compositions of the sub-units. In this study, we incorporate samples from this study to expand the geochemical dataset, and permit further comparisons to the distal counterparts. Eruption deposits preceding Aso-3 are also preserved in the proximal outcrops, although detailed stratigraphic and geochemical analysis to date has been limited. To address this, we re-investigated a key exposure at Johoku Town, located ∼24 km east-northeast of the caldera wall (32.97441° N, 131.40409° E). The logged stratigraphy at the outcrop is presented in Fig. 3.

The outcrop in Johoku Town preserves a succession of pumice lapilli and ash fall deposits separated by palaeosoils beneath the Aso-3 deposits. The lowermost exposed unit is a thin (10 cm) pumice lapilli fall unit named Aso-R, which is bracketed by two well-developed paleosols (Fig. 3). Stratigraphically above Aso-R, the Aso-OPQ eruption package is identified, which contains several sub-units with no evidence for temporal breaks. Of these, Aso-Q is the lowermost unit and is comprised of a 6 cm thick black coloured ash fall layer. Aso-P is a 55 cm thick well-sorted, clast supported pumice lapilli fall deposit, and Aso-O consists of 36 cm of alternating white ash and pumice lapilli layers. A ∼80 cm thick paleosol/tephric soil overlies the Aso-OPQ eruption package. Aso-U is located within the paleosol and is approximately 8 cm in thickness. Stratigraphically above, the 3W, 3A, and 3B phases of the Aso-3 eruption are clearly observed, and were sampled and analysed for this study. Aso-R and Aso-OPQ eruption units are also preserved at Kariono (∼9.5 km further west from Johoku; 32.99072° N, 131.30055° E). At this site, the stratigraphic relationship with the older pyroclastic flow deposits of the Kuju–Miyagi eruption (Kuju volcano) are observed, where the latter is found at the base of the exposure, below the Aso-R and a paleosol.

The Aso-2 succession is also composed of several subunits, named Aso-2A and -2B and Aso-2T (in ascending order; Ono and Watanabe, 1974; Fujii et al., 2001; Tajima et al., 2017). As highlighted, proximal glass datasets for the Aso-2 caldera-forming event are also limited, partly due to the lack of exposure and that many of the outcrops are welded and unsuitable for glass shard analysis. PCD deposits of Aso-2A (sample ITJ16) and 2B (ITJ15) were identified north of Aso, near Ichinomiya-Teno (32°59′47″N, 131°7′51″E). An ash fall deposit relating to the terminal phase (i.e., Aso-2T; sample ITJ332) was also sampled at Johoku Town, where it occurs below Aso-P.

### 2.2 Distal sites

To assess the wider dispersal and preservation of Aso-derived tephra during MIS 6–5e, we targeted two distal archives: Lake Suigetsu (SG06/SG14 core) situated in central Honshu, and an IODP marine core U1427A from the Sea of Japan (Fig. 1). These sites are strategically located within the dispersal footprint of the Aso-3 eruption (Machida and Arai, 2003, Fig. 1). In the Lake Suigetsu SG06 core, Aso-3 was not previously identified as a visible layer (Smith et al., 2013), and while the SG14 core spans the relevant time interval (>120 ka), no systematic investigation of the older tephrostratigraphy had been conducted. Similarly, although marine core U1427A contained no visible tephra between MIS 6 and 5e (Sagawa et al., 2018), its location made it a prime candidate for cryptotephra investigations.

#### 2.2.1 Lake Suigetsu core (SG06/SG14)

Lake Suigetsu is located in central Honshu (35°35′N, 135°53′E) and part of the Mikata Five Lakes system. Its formation is tectonic in nature and has a surface area of 4.3 km^2^, a diameter of ∼2 km, and a maximum depth of 34 m. The lakes are situated within a small, forested catchment dominated by warm-temperate mixed vegetation and surrounded by Palaeozoic hills reaching up to 400 m in elevation (Nakagawa et al., 2005). The main inflow is the Hasu River (∼10 km in length), which enters via Lake Mikata and flows through the shallow Seto Channel (∼2 m deep) before reaching Lake Suigetsu. Due to this restricted connection, Suigetsu receives limited detrital input from the catchment and typically only during high-energy hydrological events such as flooding.

The lake has been cored numerous times in the last three decades (see Nakagawa et al., 2012) with the SG06 and SG14 cores of relevance to this study. The SG06 core was recovered in 2006, comprising a composite sequence of approximately 73.2 m, assembled from four overlapping boreholes (A–D) spaced ∼20 m apart (Nakagawa et al., 2012). In 2014, a subsequent coring campaign was carried out ∼320 m east of SG06, extracting four new boreholes (E–H) that together form the SG14 master sequence. This SG14 sequence extends 25 m deeper than SG06 to ∼98 m and terminates at a basal gravel layer. The SG14 composite (correlation model: 28. Feb. 2019) is precisely correlated to the SG06 master core (correlation model: 06. Apr. 2020) using 361 shared stratigraphic marker layers (including visible tephras) allowing transfer of the high-precision SG06 age-model to SG14. The SG14 sequence extends to >160 ka, and was used for this investigation due to the continuous high-resolution sedimentation across MIS 6 and MIS 5e, which are the focus of this study.

The Lake Suigetsu cores are the longest continuous varved record of the Quaternary and the sequence has been dated with exceptional precision for the last 50 kyr (Schlolaut et al., 2012, 2018). More than 800 macrofossils have been radiocarbon dated from the SG06 core (Bronk Ramsey et al., 2012, 2020; Staff et al., 2011, 2013; Ramsey et al., 2020), and this dataset contributed significantly to the development of the international radiocarbon calibration curves (IntCal13 and IntCal20; Reimer et al., 2013, 2020). For the deeper sections (beyond the radiocarbon limit and of most relevant here), age estimates are produced using a *P_Sequence* deposition model in OxCal (Bronk Ramsey et al., 2012), incorporating a ^40^Ar/^39^Ar age of 86.4 ± 0.6 ka (1σ) for the Aso-4 tephra at 49.228 m CD in SG14 (Albert et al., 2019). As such, the uncertainties increase within the older sediments further from this constraint. A second age-model generated by Francke et al. (2025) is also utilised in this study, which uses an anchor point at the position of the peak in *Cryptomeria* pollen concentrations (i.e., the MIS 5e peak) in SG06 and marine core MD01-2421.

The high-resolution Lake Suigetsu sediments contain a host of climatically sensitive proxies that have enabled detailed reconstructions of climatic and environmental change spanning the 200 ka (see Nakagawa et al., 2012). The pollen and diatom reconstructions generated using the SG06 sediments are of particular relevance to this study. These sedimentary and proxy data (*Cryptomeria* and *Quercus-E* pollen percentage and counts, *Thalassiosira spp.+Cymatotheca* diatom flux) are reported in Francke et al. (2025) and Nakagawa et al. (2012, 2021).

More than 35 visible ash layers have been identified in the Suigetsu cores to date (Smith et al., 2011, 2013; McLean et al., 2016, 2018; Albert et al., 2018, 2019; Vineberg et al., 2024), most of which originate from eruptions at calc-alkaline to high-K calc-alkaline arc volcanoes situated southwest of Suigetsu and were dispersed via the prevailing westerly winds. The lake also contains >70 cryptotephra layers within the sequence, which have preserved eruptions from more distal sources and lower magnitude events, including some from Aso volcano (McLean et al., 2018, 2020a, 2020b, 2022; Albert et al., 2024; Vineberg et al., 2024, 2025). The Aso-derived ash layers <120 ka preserved in the Lake Suigetsu sequence are summarised and correlated by Vineberg et al. (2025), and this study verified that Aso-3 was not preserved in the sediments in this timeframe.

#### 2.2.2 Sea of Japan marine core (U1427A)

Marine core U1427A was recovered from the south-central Japan Sea (35°57.92′N, 134°26.06′E; 330 m water depth) during Integrated Ocean Drilling Program (IODP) Expedition 346 “Asian Monsoon” in 2013. The focus of the expedition was to investigate the evolution of the East Asian Monsoon system on orbital to millennial timescales. The sediments at Site U1427A are composed predominantly of bioturbated, homogeneous silty clay, interspersed with multiple tephra layers ranging from a few centimetres to over 0.5 m in thickness. Several additional cores were recovered during the expedition, including Site U1429A located in the northern East China Sea (Fig. 1; 31°37.04′N, 128°59.85′E).

Sagawa et al. (2018) developed a composite stratigraphy for Sites U1427A and U1429A based on major element glass chemistry, grain morphology, and heavy mineral assemblages, identifying 18 tephra layers that they correlated across the two cores. Using this integrated tephrostratigraphic framework together with benthic foraminiferal δ^18^O records, they established an orbital-scale age model for Site U1427A spanning the past ∼1.1 Myr. Their benthic foraminiferal δ^18^O records also revealed a key regional distinction: during glacial periods, the semi-enclosed Japan Sea exhibits negative δ^18^O anomalies at U1427A, contrasting with the conventional positive glacial peaks observed at U1429B in the East China Sea. This inversion is thought to reflect freshwater stratification and reduced oceanic exchange through the Tsushima and Tsugaru Straits, which alter the isotopic signal (Oba et al., 1991; Kido et al., 2007; Yokoyama et al., 2007). Compounding this, large fluctuations in the carbonate compensation depth (CCD) affect the preservation of calcareous microfossils, causing issues with the isotope stratigraphy in the Japan Sea. To overcome these challenges, tephra layers, particularly those with well-constrained ages and widespread dispersal, have been used as key tie-points to synchronise δ^18^O records between sites. Although no visible tephra of Aso-3 was identified in U1427A, a 27 cm thick unit was present in U1429A at a depth of 69.83 m (Sagawa et al., 2018).

## 3 Methods

### 3.1 Sampling proximal deposits

Representative samples of the pumice and ash were collected from the proximal exposures of the Aso-3 sub-units and the preceding events at Johoku Town and Kariono (Aso-R, Aso-OPQ, Aso-U, Aso-2; Fig. 3). Further details relating to the samples are included in the Supplementary Material. Bulk material (pumice clasts and ash) were crushed and wet sieved, removing the fraction <25 μm. Samples were oven dried at ∼60 °C and mounted in Struers Epoxy resin. The surfaces of the resin stubs were manually sectioned using silicon carbide papers to expose the shards, polished, and carbon coated for chemical analyses.

### 3.2 Sampling the cores (Lake Suigetsu and U1427A)

**Fig. 4:**
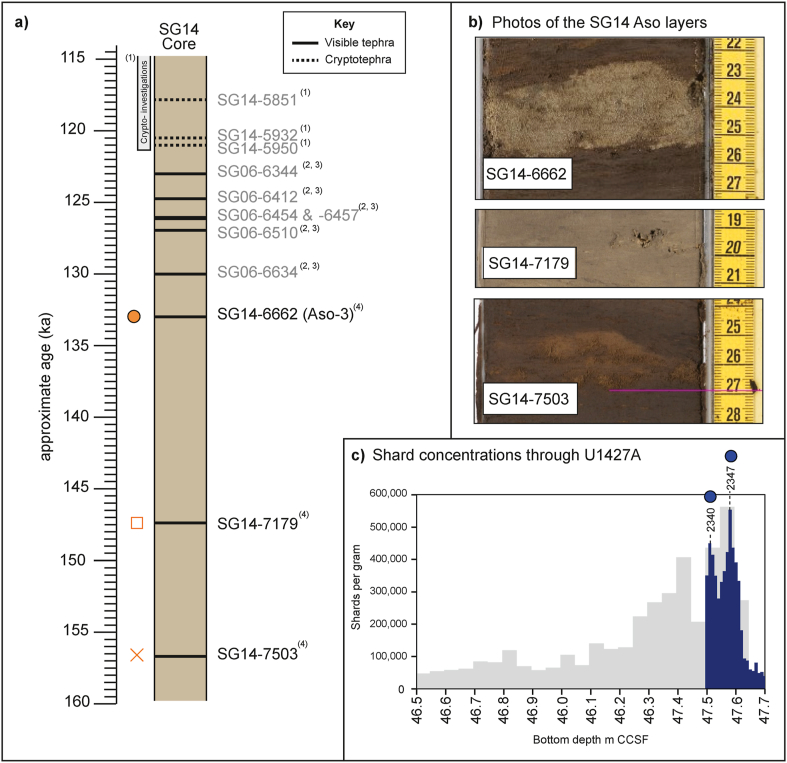
(a) Schematic of the Lake Suigetsu (SG14) tephrostratigraphy spanning ∼160–115 ka and extent of previous cryptotephra investigations by Vineberg et al. (2024). Bold lines show the position of visible tephra layers, and dotted lines show the position of non-visible (cryptotephra) layers. Tephra identifications and their correlations are published in: (1) Vineberg et al. (2024), (2) Smith et al. (2013), (3) Albert et al. (2019). (b) Images of the visible ash layers, all associated with eruptions of Aso, that are newly identified in the Lake Suigetsu (SG14) sediments - these are not preserved in SG06 (Smith et al., 2013). The SG14 tephra are named using their composite depth and their stratigraphic positions are provided in the Supplementary Material. (c) Spiked glass shard concentrations in the Sea of Japan marine core U1427A between 46.5 and 47.7 m Core Composite Depth below Seafloor (CCSF; patched version D_rev20170310). Grey bars represent low-resolution (5 cm) samples, while blue bars show high-resolution (1 cm) samples. The peaks of glass shards geochemically analysed were from samples ST2340 and ST2347 (core section 6H-5W). Orange and blue symbols in (a) and (c) correspond to those used in subsequent geochemical biplots. (For interpretation of the references to colour in this figure legend, the reader is referred to the Web version of this article.)

Visible tephra layers preserved in the Lake Suigetsu (SG14) sediments that are dated >120 ka were logged and sampled. This follows on from the lowest extent of cryptotephra investigations conducted by Vineberg et al. (2024), which reached 59.79 m CD in SG14 (i.e., 120 ka; Fig. 4). The glass shards were wet-sieved through a 25 μm mesh and mounted and polished in Struers Epoxy resin.

A detailed cryptotephra investigation was performed on core U1427A, targeting the sediments spanning the MIS 6 to 5e transition (i.e., 46.5–47.7 m Core Composite Depth below Seafloor (CCSF); Fig. 4). Since the focus was to locate Aso-3 in the core, the existing age-model was used to select the relevant section for cryptotephra analysis. The core was contiguously sub-sampled at 5 cm resolution across this interval and samples were processed using a density extraction in order to locate peaks in glass shard concentrations. Where elevated shard concentrations were detected, higher-resolution sampling at 1 cm intervals was performed to more precisely locate the peak. Sample processing followed the methods of Blockley et al. (2005) and Albert et al. (2024), with *Lycopodium* spore tablets added to calculate shard concentrations. The concentrations were quantified using the standard spike-counting equation of Payne and Gehrels (2010), and are expressed as shards per gram of dry sediment.

### 3.3 Major and trace element glass analysis

Major and minor element compositions of individual glass shards were obtained for the proximal comparative samples and distal tephra layers. These were measured using a: (1) JEOL-8600 and (2) JXA-8200 wavelength-dispersive electron microprobe at the Research Laboratory for Archaeology and History of Art (RLAHA), School of Archaeology, University of Oxford (as specified in the Supplementary Material). Electron Microprobe Analysis (EPMA) used an accelerating voltage of 15 kV, beam current of 6 nA and 10 μm-diameter beam. Peak counting times were 12 s for Na, 50 s for Cl, 60 s for P, and for 30 s for all other elements. All Fe is assumed to occur as FeO and is labelled as FeOt. The electron microprobes were calibrated using a suite of mineral standards. The accuracy and precision of these data were assessed using analyses of the MPI-DING reference glasses (Jochum et al., 2006), which were run as secondary standards. Analyses of these secondary standards lie within ±1 standard deviation of the preferred values and are presented in the Supplementary Material. All these data were filtered to remove non-glass analyses (e.g., minerals and microlites), and those with low analytical totals <93 %. The raw values were normalised (to 100 %) for comparative purposes and to account for variable glass hydration, and are presented as such in all tables and figures.

Trace element compositions of the glass shards were measured by laser ablation inductively coupled plasma mass spectrometry (LA-ICP-MS) at: (1) the Volcano and Earth's Interior Research Center, Institute for Marine Geodynamics, Japan Agency for Marine-Earth Science and Technology (JAMSTEC), and (2) the Department of Earth Sciences, Royal Holloway, University of London (RHUL) (as specified in the Supplementary Material). At JAMSTEC, the analytical equipment used include the deep-ultraviolet (200 nm) femtosecond laser ablation system (DUV-FsLA) of OK-Fs2000 K (OK Laboratory, Tokyo, Japan) connected to the modified high-sensitivity sector field ICP-MS of Element XR (Thermo Scientific, Bremen, Germany). All analyses used a 25 μm crater diameter and depth, and conditions followed those reported by Kimura and Chang (2012). Ten major elements including P_2_O_5_ and 33 trace elements were analysed for each sample, and were also run alongside several MPI-DING reference glasses (Jochum et al., 2006), and the BHVO-2 G standard provided by the United States Geological Survey. Accuracies of the BHVO-2 G glass analyses are typically <3 % for most elements, <5 % for Sc, Ga, Sm, Eu, Gd, U and <10 % for Ni, Cu, Lu. The laser-ablation system at RHUL was an Agilent 8900 triple quadrupole ICP-MS (ICP-QQQ) coupled to a Resonetics 193 nm ArF excimer laser-ablation system. Analytical procedures and data reduction (Microsoft Excel) methods followed those by Tomlinson et al. (2010). MPI-DING glasses (StHs6/80-G and ATHOG-1; Jochum et al., 2006) were analysed alongside the tephra deposits to monitor the accuracy. The reference samples along with the full dataset are provided in the Supplementary Material.

### 3.4 Age-depth modelling

The age estimates for the newly identified Lake Suigetsu tephra in SG14 are produced using a P_Sequence deposition model in OxCal (Bronk Ramsey et al., 2012), which extends the age model from the radiocarbon limit (Bronk Ramsey et al., 2012) by incorporating a^40^Ar/^39^Ar age of 86.4 ± 0.6 ka (1σ) for the Aso-4 tephra at 49.228 m CD in SG14 (Albert et al., 2019). Correlation and composite depth modelling is performed using the LevelFinder software package (v7.7.1; http://polsyems.rits-palaeo.com) which applies linear interpolation between marker layers to determine composite depths (CD) and event-free depth (EFD) values for tephra horizons. Further details of the molleding depths as well as the SG06/SG14 CD and EFD models are provided in the Supplementary Material.

The second age-depth model developed by Francke et al. (2025) is also applied in this study. This model extends the Lake Suigetsu chronology beyond the radiocarbon limit by aligning a prominent peak in Cryptomeria pollen concentration (interpreted as representing the MIS 5e climatic optimum) between Lake Suigetsu (SG06 core) and marine core MD01-2421 (offshore in the Pacific Ocean). The Cryptomeria peak occurs at ∼52.498 m composite depth (CD) in core SG06, with a corresponding feature in MD01-2421 at 32.01 m depth (Francke et al., 2025).

The age-depth model for IODP Site U1427 is based on the updated composite depth scale “U1427_patched_CCSF-D_rev20170310”. For Site U1429B, we utilise the revised Bayesian age model presented in Francke et al. (2025), which integrates 20 radiocarbon dates from benthic foraminifera and four tephra layers correlated to the Lake Suigetsu record (Sagawa et al., 2018; Smith et al., 2013). This updated model was used to refine the age estimate for the Aso-3 tephra preserved as a visible deposit in the sequence.

## 4 Results

### 4.1 Proximal glass compositions

#### 4.1.1 Aso-3 proximal deposits

**Fig. 5:**
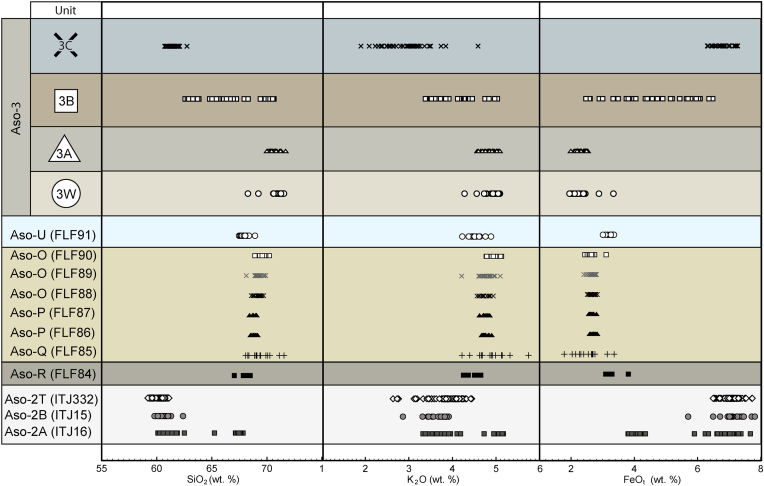
Stratigraphic positions and associated glass compositions of the proximal Aso-3 (3W, 3A, 3B, and 3C), pre-Aso-3 units (Aso-R, OPQ and U from Johoku Town (FLF84-90) and Aso-2 (ITJ332; ITJ15, ITJ16), showing variation in major element concentrations (SiO_2_, K_2_O, and FeOt). Plot symbols are used in other geochemical figures. Additional major element data (EPMA) and trace element data (LA-ICP-MS) for the proximal samples analysed are available in the Supplementary Material.

**Fig. 6:**
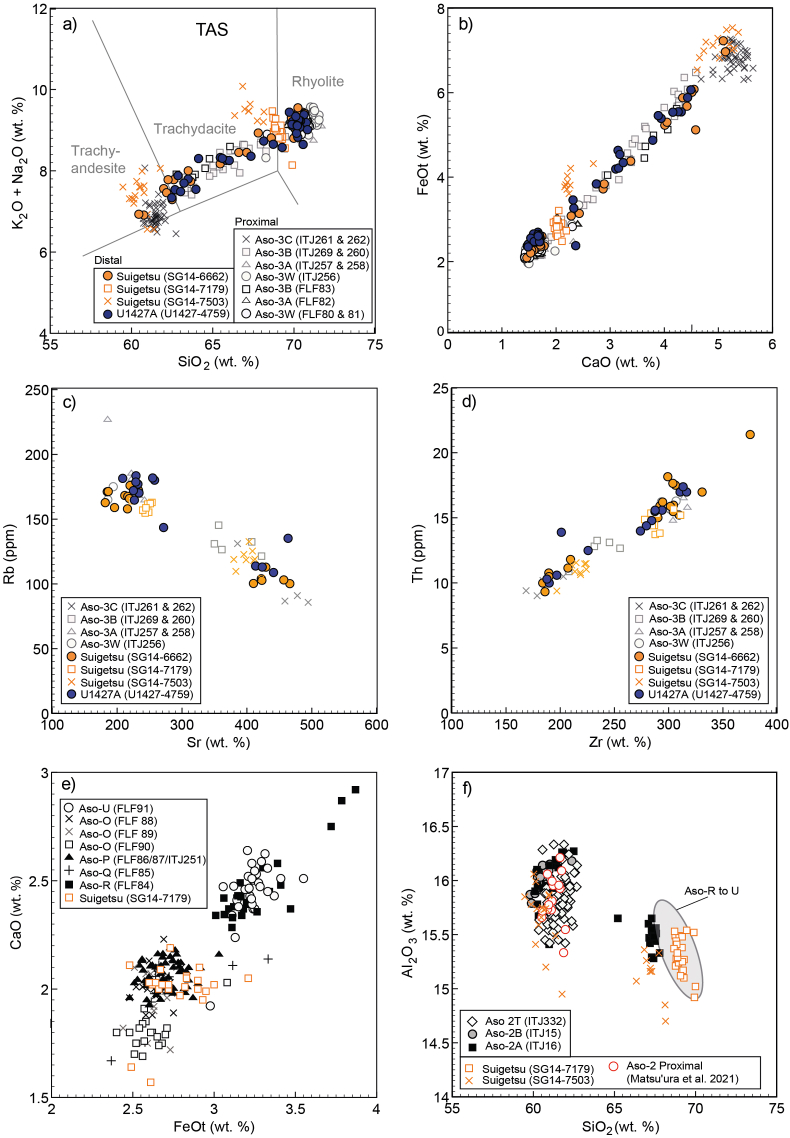
Major element glass compositions of visible tephra layers SG14-6662, SG14-7179, and SG14-7503 (orange symbols), newly identified in the Lake Suigetsu (SG14; 160–120 ka) record and cryptotephra U1427A-4759 (Sea of Japan). (a–d) Major element glass geochemistry of these distal tephras compared with proximal glass compositions from the Aso-3 eruption (white and grey symbols). (e–f) Bivariate plots used to compare SG14-7179 and SG14-7503 with other Aso units (Aso-R, OPQ and U from Johoku Town) and Aso-2 (Aso-2A, 2B and 2T), highlighting their compositional similarities and distinctions. The Total Alkali–Silica (TAS) classification in (a) follows Le Bas et al. (1986). (For interpretation of the references to colour in this figure legend, the reader is referred to the Web version of this article.)

**Fig. 7:**
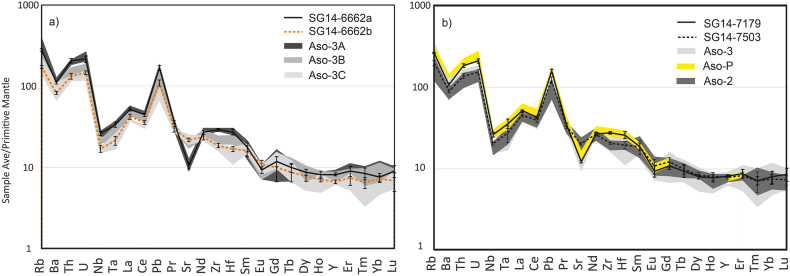
Sample volcanic glass averages normalised to primitive mantle compositions for the newly identified visible ash layers preserved in the Lake Suigetsu (SG14 core) spanning 160–120 ka. (a) Shows the average profile of SG14-6662 Population A (<65 wt % SiO_2_; *n = 11*) and B (>65 wt % SiO_2_; *n = 6*) in comparison to the proximal deposits of the Aso-3 eruption units. (b) Shows the average profile of SG14-7179 (*n = 9*) and SG14-7503 (*n = 9*) in comparison to the proximal deposits of Aso-3 (ITJ256 - ITJ162 combined), Aso-P (ITJ251) and Aso-2 (ITJ332). Primitive mantle values used for normalisation follow Sun and McDonough (1989). Error bars represent the range of the sample average. Full compositional datasets are provided in the Supplementary Material.

The major element glass compositions of the Aso-3 succession (units 3W, 3A, 3B, and 3C) for all samples are consistent with those reported by Kaneko et al. (2015) and the equivalent units at the Noga outcrop reported by Albert et al. (2019). As shown in Fig. 5, Fig. 6, Units 3W and 3A are the most silicic, with glass compositions ranging from ∼69 to 71 wt% SiO_2_, 4.5–5.0 wt% K_2_O, and 2.0–3.0 wt% FeOt. Unit 3 B displays a broader compositional range (∼62–70 wt% SiO_2_, 3.5–5.0 wt% K_2_O, 2.5–6.5 wt% FeOt), with transitional compositions bridging the more silicic (3W/3A) and more mafic (3C) end members. Unit 3C is the least evolved, with compositions ranging from ∼60 to 64 wt% SiO_2_, 2–5 wt.% K_2_O, and 6–7.5 wt% FeOt (Fig. 5). The trace element compositions also help to discriminate the Aso-3 units (Fig. 6; Fig. 7) and show enrichment in incompatible elements (Zr = 168–304 ppm; Th = 9.4–16.2 ppm), while Sr decreases from ∼477 ppm to ∼185 ppm with increasing SiO_2_ (Supplementary Material).

#### 4.1.2 Pre-Aso-3 proximal deposits

The pre-Aso-3 tephra units (Aso-R, Aso-OPQ, and Aso-U) from Johoku Town exhibit relatively homogeneous rhyolitic glass compositions and plot towards the evolved-end of the Aso-3 compositional field (Fig. 5). Most glasses fall between ∼68.5 and 70.2 wt% SiO_2_ and ∼4.6–5.1 wt% K_2_O, with low FeOt and CaO values. Their compositions align with the more silicic Aso-3 units (3W/3 A), though they are ∼1–2 wt.% lower in SiO_2_ (Fig. 6).

The stratigraphically oldest (Aso-R; FLF84) and youngest (Aso-U; FLF91) units are the least evolved and most compositionally variable, with SiO_2_ ranging from ∼67 to 69 wt%. Both exhibit slightly higher CaO and K_2_O (by ∼0.5 wt%) than the intervening Aso-OPQ tephra. Aso-OPQ is generally compositionally consistent across all samples. Of these, Aso-Q (sample FLF85) spans the broadest SiO_2_ range (∼68–71 wt%), while Aso-P (FLF86–87) averages ∼68.8 wt% SiO_2_ (Fig. 6e). Among the three samples analysed within Aso-O (FLF88–90), the uppermost (FLF90) is the most SiO_2_-rich and compositionally restricted.

Trace element compositions for Aso-P (ITJ251 Johoku Tunnel; Fig. 7) shows the glasses contain elevated incompatible element concentrations (Zr = 308.5 ± 22.6 ppm; Th = 16.9 ± 1.2 ppm; U = 5.2 ± 0.4 ppm), high Rb (∼180 ppm) and Ba (∼900 ppm), and lower Sr (∼270 ppm). Although the trace element pattern closely follows the overall incompatible element trend seen in Aso-3 and Aso-2, they display slightly elevated concentrations for several elements, particularly the middle to heavy rare earth elements (e.g., Y, Er, Yb).

#### 4.1.3 Aso-2 proximal deposits

The glasses within the stratigraphically oldest unit of the Aso-2 eruption (i.e., Aso-2A; ITJ16) are bimodal in composition, comprising a lower-SiO_2_ group (∼61.2 wt%) and a higher-SiO_2_ group (∼67.4 wt %) (*n = 38*; Fig. 5). No intermediate compositions were observed between these two groups. In contrast, unit Aso-2B contains glass shards forming a single compositional population with ∼60.6 wt % SiO_2_ (*n = 22*). The Aso-2T deposit sampled from Johoku Town (ITJ332) consists of trachy-andesitic glass shards (Fig. 5, Fig. 6). These glasses are relatively homogeneous, ranging from 60.3 to 62.7 wt% SiO_2_, 2.6–4.4 wt% K_2_O, and 6.3–7.7 wt% FeOt (n = 67). Their compositions are most consistent with those reported by Matsu-ura et al. (2021) (Fig. 6f) and are notably ∼1 wt% higher in K_2_O than the least-evolved component of the Aso-3 eruption (i.e., unit 3C; Fig. 7b).

### 4.2 Distal glass compositions

#### 4.2.1 Lake Suigetsu (SG14 core)

Three visible ash layers were identified in the sediments of the Lake Suigetsu (SG14) core between ∼60 m CD and the base of the core. These are named using their SG14 composite depths (from youngest to oldest) as SG14-6662 (∼3.0 cm thick), SG14-7179 (∼0.5 cm), and SG14-7503 (∼1.5 cm) (Fig. 4; Table SM1). The ages of the SG14 tephra layers were derived using the Bayesian age model outlined in Section 3.4 and the stratigraphic positions as listed in the Supplementary Material. These produced estimates of ∼132.8 ± 1.6 ka for SG14-6662, ∼147.4 ± 4.9 ka for SG14-7179, and ∼156.7 ± 6.4 ka for SG14-7503 (1σ).

Although the SG06 core sediments extend to the depth of SG14-6662, this tephra was not identified as a visible layer. This is likely due to the shallower lake depth at the SG06 core site, which lies ∼320 m west of SG14. Such conditions at the SG06 location are inferred from sedimentological and biogeochemical proxies, which indicate that Lake Suigetsu was still undergoing tectonic formation during the late MIS 6 period (ca. 140–130 ka), and that stable lacustrine conditions had not yet been fully established across the entire current footprint of the lake at that time (see Section 5.3; Francke et al., 2025). Future work on the SG06 sediments will likely identify SG14-6662 as a cryptotephra horizon.

The glass compositions of the three Lake Suigetsu tephra are presented in Table 1 and Fig. 6, Fig. 7. All three tephra layers exhibit HKCA glass compositions characteristic of Aso caldera tephra including total alkalis (K_2_O + Na_2_O) > 8 wt %, and ∼15 wt % Al_2_O_3_ (Table 1; Fig. 5). Trace element signatures show enrichment in large ion lithophile elements (LILE, e.g., Rb) and high field strength elements (HFSE, e.g., Th, U, Zr), also typical of Aso-derived tephra (Fig. 6).

**Table 1:** Major and trace element glass compositions (normalised) of the newly identified visible ash layers preserved in the Lake Suigetsu (SG14) core between 160 and 120 ka and the cryptotephra layer U1427A-4759 (Sea of Japan). Groups a (i.e., SG14-6662a and U1427a) include analyses with >65 wt. % SiO_2_ and Groups b are those with <65 wt. %. Raw datasets and secondary standards are included in the Supplementary Material.

	SG14-6662a		SG14-6662b		SG14-7179		SG14-7503		U1427A-4759a		U1427A-4759b	
wt. (%)	Avg.	±1 σ	Avg.	±1 σ	Avg.	±1 σ	Avg.	±1 σ	Avg.	±1 σ	Avg.	±1 σ
SiO_2_	69.32	1.54	62.20	1.07	69.06	0.35	62.97	3.37	69.32	1.82	63.25	0.64
TiO_2_	0.67	0.13	0.93	0.05	0.73	0.04	1.02	0.22	0.63	0.10	0.91	0.06
Al2O_3_	15.27	0.21	16.16	0.21	15.28	0.16	15.47	0.37	15.47	0.43	16.13	0.10
FeOt	2.71	0.66	5.94	0.74	2.78	0.17	5.96	1.59	2.72	0.77	5.43	0.40
MnO	0.10	0.04	0.14	0.04	0.11	0.03	0.13	0.05	0.11	0.02	0.15	0.01
MgO	0.65	0.27	1.96	0.28	0.73	0.05	1.79	0.81	0.67	0.28	1.75	0.18
CaO	1.95	0.57	4.52	0.39	2.00	0.14	4.03	1.37	1.95	0.58	4.16	0.28
Na_2_O	4.29	0.16	4.15	0.24	4.29	0.14	4.09	0.44	4.29	0.45	4.23	0.21
K_2_O	4.74	0.35	3.34	0.18	4.76	0.13	4.05	0.72	4.63	0.33	3.47	0.13
P_2_O_5_	0.13	0.09	0.52	0.07	0.15	0.03	0.38	0.16	0.12	0.09	0.43	0.07
Cl	0.16	0.05	0.12	0.03	0.11	0.03	0.09	0.03	0.10	0.03	0.08	0.02
*n =*	*18*		9		22		25		34		7	
	Oxford EPMA		Oxford EPMA		Oxford EPMA		Oxford EPMA		Oxford EPMA		Oxford EPMA	

	SG14-6662a		SG14-6662b		SG14-7179		SG14-7503		U1427A-4759a		U1427A-4759b	
(ppm)												
Rb	171.39	13.51	122.70	8.65	174.90	5.00	167.21	30.67	172.56	11.96	117.16	11.83
Sr	276.31	55.86	446.58	35.90	280.20	23.10	312.87	88.79	237.56	18.72	435.78	21.85
Y	32.17	1.31	28.48	1.50	34.10	0.90	33.29	2.78	32.47	2.72	28.40	2.21
Zr	279.13	23.12	199.54	15.99	295.30	11.10	279.22	52.06	285.56	32.94	200.71	17.56
Nb	16.83	1.20	12.22	1.31	17.90	0.60	17.24	2.86	16.53	2.32	12.20	0.94
Ba	798.49	52.69	635.68	40.50	851.30	36.00	795.88	109.77	835.66	41.22	620.59	49.23
La	34.53	1.45	28.47	2.17	36.40	1.10	35.86	4.23	35.15	1.88	28.27	2.30
Ce	76.21	2.88	64.07	4.90	79.30	2.40	78.84	8.72	77.51	4.03	61.68	5.54
Pr	8.69	0.27	7.51	0.58	9.00	0.40	9.07	0.85	8.93	0.50	7.33	0.68
Nd	35.78	1.20	31.81	2.31	17.90	0.60	37.00	2.93	35.68	1.96	30.87	3.05
Sm	7.41	0.25	6.86	0.41	7.80	0.30	7.84	0.61	7.16	0.45	6.96	0.17
Eu	1.52	0.07	1.61	0.07	1.60	0.10	1.64	0.08	1.45	0.13	1.58	0.16
Gd	6.09	0.31	5.85	0.32	6.30	0.50	6.56	0.29	6.16	0.49	5.55	0.39
Dy	5.77	0.28	5.24	0.30	6.00	0.30	5.99	0.43	5.70	0.47	5.26	0.34
Er	3.40	0.25	3.02	0.17	3.60	0.20	3.59	0.33	3.43	0.34	2.99	0.24
Yb	3.53	0.20	2.97	0.21	3.70	0.30	3.69	0.36	3.54	0.32	3.08	0.27
Hf	7.33	0.70	5.30	0.39	7.60	0.50	7.32	1.32	7.76	1.03	5.36	0.60
Ta	1.18	0.09	0.86	0.09	1.20	0.10	1.23	0.21	1.15	0.20	0.83	0.08
Th	15.01	1.39	10.68	0.82	15.10	0.80	14.83	2.81	15.54	1.27	10.83	1.13
U	4.50	0.37	3.23	0.23	4.40	0.30	4.47	0.85	4.65	0.59	3.16	0.40
*n =*	*15*		12		9		10		10		4	
	RHUL LA-ICP-MS		RHUL LA-ICP-MS		RHUL LA-ICP-MS		RHUL LA-ICP-MS		RHUL LA-ICP-MS		RHUL LA-ICP-MS	

	SG14-6662a		SG14-6662b		SG14-7179		SG14-7503	
wt. (%)	Avg.	±1 σ	Avg.	±1 σ	Avg.	±1 σ	Avg.	±1 σ
SiO_2_	69.51	1.27	60.89	1.15	68.07	0.86	59.23	0.83
TiO_2_	0.64	0.09	0.98	0.05	0.72	0.02	1.16	0.04
Al_2_O_3_	15.28	0.73	16.61	0.28	15.40	0.19	16.14	0.11
FeOt	2.50	0.35	6.27	0.58	2.72	0.13	7.22	0.35
MnO	0.10	0.01	0.15	0.00	0.11	0.00	0.15	0.01
MgO	0.54	0.10	2.12	0.23	0.72	0.03	2.28	0.18
CaO	1.61	0.36	4.66	0.45	2.26	0.94	5.13	0.31
Na_2_O	4.93	0.45	4.62	0.19	5.29	0.27	4.56	0.13
K_2_O	4.79	0.34	3.10	0.15	4.55	0.14	3.48	0.19
P_2_O_5_	0.10	0.02	0.41	0.09	0.16	0.00	0.66	0.07
*n =*	*11*		6		9		9	
	JAMSTEC LA-ICP-MS		JAMSTEC LA-ICP-MS		JAMSTEC LA-ICP-MS		JAMSTEC LA-ICP-MS	

(ppm)								
Rb	171.39	12.88	103.90	4.69	159.27	3.62	118.82	8.28
Sr	195.81	48.09	434.56	21.94	243.73	9.48	401.49	13.89
Y	35.62	2.02	28.54	1.56	35.12	1.06	33.46	1.76
Zr	309.31	23.26	194.24	11.14	290.87	11.09	215.95	8.56
Nb	17.61	1.71	29.50	2.29	17.28	1.24	13.24	0.49
Ba	719.07	62.22	546.17	26.42	717.95	31.35	595.22	28.69
La	34.06	2.54	27.33	1.99	33.53	1.04	29.68	1.71
Ce	76.14	7.78	59.56	3.01	72.63	1.87	65.35	3.77
Pr	8.91	0.43	7.28	0.64	8.56	0.34	8.20	0.62
Nd	34.14	2.11	29.50	2.29	33.43	2.00	33.00	0.99
Sm	7.26	0.89	6.49	0.96	7.65	0.73	7.53	1.50
Eu	1.37	0.23	1.67	0.21	1.45	0.24	1.66	0.35
Gd	6.41	0.87	5.46	1.85	5.92	0.65	6.57	0.91
Dy	6.06	0.82	5.18	0.64	5.39	0.34	5.48	0.49
Er	4.01	0.57	3.25	0.63	3.82	0.48	3.61	0.30
Yb	3.39	0.39	3.18	0.32	3.52	0.55	3.26	0.45
Hf	7.90	0.84	4.76	0.33	7.38	0.76	5.52	0.60
Ta	1.27	0.14	0.79	0.10	1.33	0.18	1.00	0.24
Th	16.78	1.77	10.58	0.87	14.75	0.73	10.95	0.68
U	4.51	0.59	2.96	0.14	4.29	0.29	3.12	0.25
n =	11		6		9		9	
	JAMSTEC LA-ICP-MS		JAMSTEC LA-ICP-MS		JAMSTEC LA-ICP-MS		JAMSTEC LA-ICP-MS	

#### 4.2.2 SG14-6662 glass compositions

SG14-6662 (∼133 ka) is compositionally heterogenous, containing rhyolitic, trachy-dacite and trachy-andesite volcanic glass shards (Fig. 6a). Compositions range from 60.5 to 70.7 wt % SiO_2_, 3.0–5.3 wt % K_2_O, and 2.1–7.2 wt % FeOt (*n = 27*; Table 1; Supplementary Material). This encompasses and overlaps with the full compositional range reported for the Aso-3 proximal units (Kaneko et al., 2015, Fig. 5). The more silicic group (i.e., those with >65 wt % SiO_2_; named Population A), contains glasses with relatively lower Al_2_O_3_ (by ∼1 wt %), lower CaO (∼2 wt %) and elevated K_2_O (∼1 wt%) compared to the more mafic glasses (i.e., those with <65 wt % SiO_2_; named Population B). Trace element concentrations (Table 1; Fig. 6; Fig. 7) are equally hetrogneous showing a wide range in level of enrichment in incompatible elements (e.g., Th = 9–20 ppm; Zr = 180–330 ppm), with Sr becoming more depleted with increasing Th content. Consequently, the Population A glasses display lower Sr content than the Population B glasses. The more evolved compositions of Population A are more consistent with proximal Units 3W (fall) and 3A (flow; i.e., the earliest Aso-3 eruptive phases; Kaneko et al., 2015), while the trachytic to trachy-andesitic glasses match subsequent Units 3B and 3C flow phases (Fig. 5, Fig. 6).

#### 4.2.3 SG14-7179 glass compositions

SG14-7179 (∼147.5 ka) volcanic glasses are homogeneous rhyolites (SiO_2_ = 69.1 ± 0.4 wt %; *n = 22*), lacking the transitional or mafic compositions observed in SG14-6662 (Table 1; Fig. 6). They are compositionally distinct from the silicic glass population of SG14-6662 (Population A) at both major and trace element level (Fig. 6; Fig. 7). SG14-7179 glasses exhibit lower SiO_2_ (i.e., <68 wt%) and elevated FeOt and CaO compared to Population A of SG14-6662. SG14-7179 glasses are also less enriched in incompatible elements such as Zr (270 vs. 440 ppm), Nb (60 vs. 85 ppm), Th (23 vs. 37 ppm), and U (6.5 vs. 9.5 ppm), and also have lower Rb and Ba (Fig. 6; Fig. 7). In contrast, SG14-7179 retains higher Sr content (120 ppm vs. 47 ppm in SG14-6662A), consistent with a less evolved source.

#### 4.2.4 SG14-7503 glass compositions

SG14-7503 (∼157 ka) glasses are compositionally bimodal, with mafic trachy-andesite and trachy-dacite populations (Table 1; Fig. 6). The trachy-andesite population is characterised by low SiO_2_ (∼60.5 wt %), elevated K_2_O (∼3.5 wt %) and high FeOt (∼7.1 wt %) (*n = 16*), while the trachy-dacite population has higher SiO_2_ (∼67.5 wt%), elevated K_2_O (5.0 wt %), and lower FeOt (3.9 wt %) (*n = 9*). No transitional compositions were observed within the tephra deposit. Compared to SG14-6662, SG14-7503 glasses plot on a distinctly higher SiO_2_ vs. K_2_O array, by up to ∼1.5 wt% K_2_O at equivalent SiO_2_ content. Trace element glass analysis (Fig. 6; Fig. 7) reinforce the bimodality of the SG14-7503 glasses. The trachy-dacite glasses are enriched in incompatible elements such as Zr, Nb, Th, and U, while the trachy-andesite population exhibits high FeOt and lower levels of incompatible trace element enrichment (Table 1; Fig. 6b). Compared to SG14-6662, SG14-7503 trachy-dacite glasses have lower Sr and a flatter middle REE pattern, further distinguishing them geochemically (Fig. 6b).

#### 4.2.5 Sea of Japan marine core U1427A

Elevated concentrations of glass shards, extending to ∼500,000 shards per gram of dried sediment (shards/g), were identified near the base of Section 6H-5W of marine core U1427A (Fig. 4c). Shard concentrations in the high-resolution samples, revealed two distinct peaks. The first peak is located at 47.58–47.59 m (sample ST2347), with a maximum shard count of 553,773 shards/g. A younger peak was detected between 47.51 and 47.52 m (sample ST2340), with a shard concentration of 449,539 shards/g (Fig. 4c). These peaks occur near the MIS 6–5e transition, as constrained by the δ^18^O record from U1427A (Sagawa et al., 2018), and lie below the visible SK tephra erupted from Sambe volcano (see Section 5.2).

#### 4.2.6 U1427A-4759 glass compositions

Major element compositions of glass shards from both peaks are geochemically indistinguishable (Supplementary Material), with similar relative proportions of the range of compositions, indicating they represent the same eruptive phase. We interpret the oldest peak (positioned at 47.58–47.59 m), as the primary fallout layer and the upper as reworking and focussing of the tephra on the sea floor. The primary cryptotephra layer is herein named using its composite depth as U1427A-4759.

The glass shards from U1427-4759 span a broad compositional range from trachy-andesites, through trachy-dacite to a rhyolitic end-member, with SiO_2_ = 62.5–71.8 wt%, Al_2_O_3_ = 15.0–17.0 wt%, K_2_O = 3.3–5.0 wt%, and FeOt = 2.1–6.0 wt% (*n = 40*; Table 1; Fig. 6). This compositional diversity mirrors that of the near-source Aso-3 glass compositions. Trace element analysis reveals significant compositional heterogeneity and elevated concentrations of incompatible elements, including Rb (108.3–182.7 ppm), Zr (188.4–317.3 ppm) and Th (10.0–17.4 ppm) (*n = 14*; Table 1; Fig. 7), the latter being consistent with an Aso origin. Using increasing Th as a fractionation index, we observe that Sr is behaving compatibly with decreasing concentrations (Sr = 464-209 ppm).

## 5 Discussion

### 5.1 Tephra correlations in Lake Suigetsu and U1427A

#### 5.1.1 Aso 3 (SG14–6662 and U1427A-4759)

By integrating major and trace element data with proximal stratigraphic information, we demonstrate a robust correlation between SG14-6662 (Lake Suigetsu) and U1427A-4759 (Sea of Japan) and the caldera-forming Aso-3 eruption. Both tephra layers contain glass compositions spanning the full geochemical range documented in proximal Aso-3 deposits. The occurrence of this complete compositional spectrum at two sites ∼130 km apart suggests that all eruptive phases were widely dispersed and deposited in rapid succession. SG14-6662 is the only Aso-derived tephra layer (visible or cryptic) in the Lake Suigetsu sequence older than 110 ka that exhibits this geochemical breadth. This pattern is consistent with other distal records, where the Aso-3 isochron is preserved as a single stratigraphic layer showing the same compositional diversity. The late MIS 6 age (∼133 ka) of both SG14–6662 and U1427A-4759 further supports this correlation.

Although numerous occurrences of tephra have been tentatively attributed to Aso-3, we find that only some of these occurrences are certainly linked to the Aso-3 eruption. Robust distal correlations include Spike G16 in MD01-2422 (Matsu'ura et al., 2021), G21 in U1437 (Schindlbeck et al., 2018; Matsu'ura et al., 2023), and the tephra identified in U1429B (Sagawa et al., 2018) (Fig. 1; Fig. 2). These correlations further underscore the wide regional dispersal of the Aso-3 eruption. Other reported candidates in other distal records may also correlate to Aso-3, such as the layers in Lake Biwa and Ulleung Basin (Nagahashi et al., 2004; Chun et al., 2004), based on their chronostratigraphic position, but their full compositional datasets are not available to compare to and verify the eruptions.

#### 5.1.2 Aso-OPQ and SG14-7179

Glass compositions of SG14-7179 were compared with those from pre-Aso-3 (intra-caldera) eruptions sampled in proximal exposures (Aso-R, Aso-OPQ, and Aso-U). The SG14-7179 glasses show clear compositional overlap with the Aso-OPQ eruption package, including elevated FeOt and CaO relative to Aso-3 (Fig. 6, Fig. 7). Glasses from each of the Aso-OPQ subunits are represented within SG14-7179, supporting the inference that these eruptive phases were closely spaced in time, as also indicated by the proximal stratigraphy. SG14-7179 does not compositionally overlap with any other MIS 5 or 6 tephras previously reported in Japanese distal records. Its occurrence ∼600 km from Aso caldera confirms that the other intra-caldera eruptions were also widely dispersed, and that the compositionally distinct Aso-OPQ tephra may provide a useful isochron for mid-MIS 6 (∼140 ka).

#### 5.1.3 Aso-2 and SG14-7503

The major and trace element characteristics of SG14-7503 glasses are consistent with the proximal sub-unit of Aso-2A, the bimodal Aso-2 tephra identified by Machida and Arai (2003), the BT-42 layer in Lake Biwa (Nagahashi et al., 2004) and G-18 in MD012422 (Matsu'ura et al., 2021). Aso-2 glasses follow a distinct geochemical trend, with K_2_O concentrations up to ∼1.5 wt% higher than Aso-3 at equivalent SiO_2_ contents (Nagahashi et al., 2004), meaning the most widespread component is distinctive. The UT-14.55 layer in the Uwa Basin, tentatively correlated with Aso-2 by Tsuji et al. (2018) and Matsu'ura and Ueno (2022), contains only the low-SiO_2_ glass population that matches the mafic end member of SG14-7503. Further detailed geochemical and stratigraphic work, similar to that undertaken here for the Aso-3 tephra, would help to confirm these correlations and better constrain the compositional variability of Aso-2.

### 5.2 Stratigraphic position of the Aso-3 eruption and relevance for climate studies

**Fig. 8:**
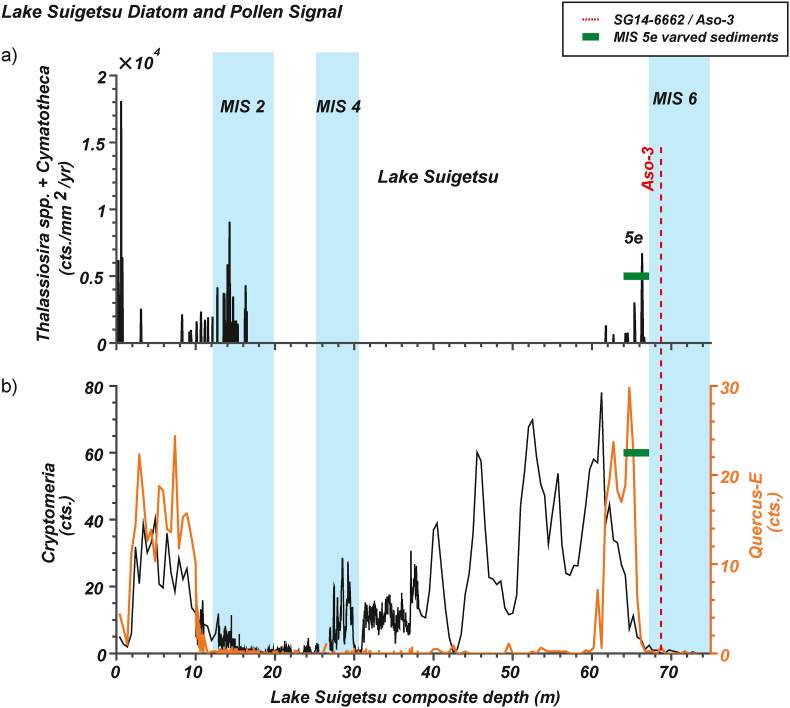
Flux of (a) marine diatom taxa Thalassiosira spp. + Cymatotheca (counts/mm^2^/year), and (b) percentages of Cryptomeria and Quercus-E pollen, recorded in the Lake Suigetsu (SG06) sedimentary sequence. The red dashed line indicates the position of the Aso-3 distal tephra layer (SG14-6662). The green bar marks the interval of laminated sediments corresponding to Marine Isotope Stage (MIS) 5e. Modified from Francke et al. (2025). (For interpretation of the references to colour in this figure legend, the reader is referred to the Web version of this article.)

**Fig. 9:**
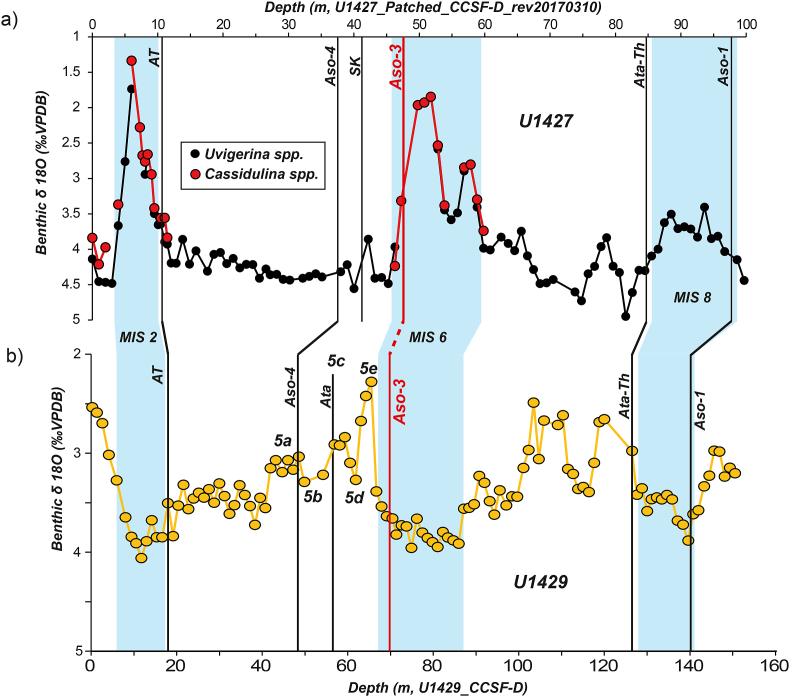
Benthic foraminiferal δ^18^O values for marine cores (a) U1427A from the Sea of Japan and (b) U1429B (data and figure modified from Sagawa et al., 2018). The position of the Aso-3 tephra is marked by a red line in U1427A (cryptotephra; this study) and U1429B (visible tephra; Sagawa et al., 2018). Other key tephra layers, including Aso-1, Ata-Th (from Ata caldera, Fig. 1), SK (from Sambe volcano), Ata, Aso-4 and the AT (from Aira caldera), are indicated by black lines. Benthic δ^18^O values were measured on *Cassidulina spp*. (red dots) and *Uvigerina spp.* (black dots), which are common deep-sea taxa and reliable recorders of bottom-water conditions. Note that δ^18^O values in the Sea of Japan are reversed relative to typical global records (see Sagawa et al., 2018 and main text). (For interpretation of the references to colour in this figure legend, the reader is referred to the Web version of this article.)

The identification of the Aso-3 tephra layer in the Lake Suigetsu (SG14-6662) and Sea of Japan marine core U1427A (U1427A-4759) allows the stratigraphic timing and position of the caldera-forming eruption to be constrained (Fig. 8; Fig. 9). In Lake Suigetsu, the Aso-3 tephra (SG14-6662) is dated to ∼133 ka (±1.6) using the Suigetsu age model. It lies just below a period of varved sedimentation (67.20–63.95 m SG06 CD) and a peak in marine diatom taxa (*Thalassiosira* spp. and *Cymatotheca*; between 50.00 and 61.77 m SG06 CD; Fig. 8a), both of which indicate lake water stratification and marine incursions between ∼129 and 121 ka (Francke et al., 2025), consistent with the MIS 5e sea-level high-stand (Lisiecki and Raymo, 2005). This interval also coincides with elevated pollen percentages of evergreen *Quercus*-E, marking the expansion of warm-temperate forest and peak summer temperatures at ∼125 ka. A delayed subsequent rise in *Cryptomeria* pollen concentrations (Fig. 8b) reflects increased monsoonal precipitation and a shift in vegetation regimes. As a moisture-demanding conifer, *Cryptomeria* typically dominates cool-temperate mixed forests in Japan, and its abundance is interpreted as a proxy for intensified summer rainfall during the latter part of peak interglacial conditions (Hope et al., 2004; Miyoshi et al., 1999).

The Lake Suigetsu SG06 core sediments between ∼140 and 130 ka are characterised by high, up to 25 % total organic carbon (TOC), and comprised of alternating peat, massive inorganic clay, and finely laminated clay (Nakagawa et al., 2012; Francke et al., 2025). Elevated TOC/TN ratios (>15) suggest dominance of terrestrial C_3_ plant material, consistent with peat-forming environments in a dynamic, semi-aquatic landscape as expected during late MIS 6. In contrast, post-130 ka sediments show a shift toward algal-derived organic matter and more stable lacustrine conditions under an interglacial climate (Francke et al., 2025).

In the marine realm, the Aso-3 cryptotephra in U1427A (U1427A-4759) and visible ash deposit in U1429A (East China Sea; Sagawa et al., 2018) are also identified within late MIS 6 sediments, prior to the MIS 5e transition (Termination II) (Fig. 9). As highlighted by Sagawa et al. (2018) and summarised in Section 3, the δ^18^O records from these two marine sites diverge due to their contrasting hydrographic regimes. The Japan Sea is a semi-enclosed basin, and during glacial low-stands, reduced exchange through the Tsushima and Tsugaru Straits leads to surface freshening and stratification. This results in reversed δ^18^O signals, with anomalously low values during glacial maxima (Oba et al., 1991; Sagawa et al., 2018). In contrast, Site U1429A, located ∼730 km southwest of U1427A, in the open East China Sea exhibits a conventional δ^18^O profile, with higher values during glacial periods reflecting increased ice volume and lower deep-water temperatures. The visible ash Aso-3 deposit reported in U1429A and can now be used as an additional anchor to link the East China Sea and Sea of Japan records, and confirms the reversed isotopic signal is also recorded during the terminal stages of MIS 6 (Fig. 9).

While the Aso-3 tephra aligns closely with the onset of Termination II in the open-marine U1429A record, its occurrence in U1427A coincides with a phase when the transition from a restricted to more fully marine Japan Sea appears largely complete. This likely reflects a rapid, threshold-driven hydrographic response to sea-level rise that is not necessarily expressed synchronously in open-ocean benthic δ^18^O records.

The updated Bayesian age-model for U1429A (Francke et al., 2025) indicates that the Aso-3 eruption was deposited at 131.0–134.0 cal yrs BP (1σ), falling in line with the modelled age of Lake Suigetsu (∼133 ka).

### 5.3 Aso-3 and considerations as a time-stratigraphic marker

The Aso-3 tephra is a regionally significant time-stratigraphic marker for correlating palaeoenvironmental and geological archives across Japan and the broader East Asian margin. Its widespread dispersal makes it one of the most spatially extensive isochrons in the region, complementing other well-established markers such as the ∼7.2 ka K-Ah (from Kikai caldera, Fig. 1), ∼30 ka AT (Aira-TN from Aira caldera), and ∼86 ka Aso-4 tephras (Machida and Arai, 2003) and allow records to be synchronised at multiple points in the last ∼135 ka. These tephras provide important chronological anchors that enable direct comparison of information recorded by disparate records. Aso-3 is particularly valuable due to its stratigraphic position near Termination II, marking the transition from the glacial conditions of MIS 6 to the interglacial onset of MIS 5e (∼135–125 ka), just prior to major regional climatic reorganisation. This period is increasingly recognised as a critical analogue for future climate change scenarios, particularly in terms of elevated polar temperatures and ice-sheet retreat, despite differences in orbital forcing and the longer-term sea-level history compared to the Holocene (Hearty et al., 2007; Grant et al., 2012; Tam and Yokoyama, 2021).

The transition from MIS 6 to MIS 5e in Japan was not characterised by a uniform or abrupt shift into interglacial conditions, but instead by dynamic, regionally asynchronous environmental changes. Notably, this interval saw significant reconfiguration of the EAM, with pronounced contrasts in precipitation and runoff between central and southwestern Japan (Tam and Yokoyama, 2021). These hydroclimatic variations were further modulated by glacio-eustatic sea-level rise, which affected coastal inundation and marine influence in low-lying inland basins such as Lake Suigetsu (Sugihara, 1970; Sagawa et al., 2018; Nakagawa et al., 2021). Nearby in Wakasa Bay (Fukui Prefecture), sea-level elevation during MIS 5e may have been between ∼60 and 115 m higher than the glacial low-stand (Tam and Yokoyama, 2021). However, due to active tectonic uplift across parts of Japan, relative sea-level expression varies considerably by region. Consequently, many reconstructions of MIS 5e sea-level changes in Japan rely on dating and correlation of uplifted marine terraces, which benefit from the presence of widespread and datable tephra layers like Aso-3. The identification of Aso-3 in both terrestrial (e.g., Lake Suigetsu, Lake Biwa) and marine environments (e.g., Sites U1427A and U1429B), and possibly in uplifted coastal sequences across Japan (Machida and Arai, 1994), underscores its potential as a valuable marker for synchronising environmental change across different depositional settings.

Nonetheless, this study highlights the need for caution when using Aso-3 as a time-stratigraphic marker. As highlighted, several distal occurrences attributed to Aso-3 in the literature lack the full compositional range of the Aso-3 eruption deposit that are observed in both proximal and distal settings. These tephra layers typically only have the rhyolitic compositions and are not found at the transition between MIS 6 and 5e. They may reflect another separate eruption from Aso, as our results suggest that multiple, compositionally distinct eruptions from Aso occurred between its major caldera-forming events, some of which have been deposited over 550 km from Aso (e.g., McLean et al., 2020a; Vineberg et al., 2025). In this study, we find a compositionally distinct visible ash layer dated to ∼147 ka in Lake Suigetsu, that shares the same glass compositions as the Aso-OPQ eruption deposits. This unit is stratigraphically situated between the Aso-2 and Aso-3 caldera-forming episodes, further illustrating the persistent explosive activity of Aso through MIS 6.

There may be other eruptions both preceding and following Aso-3, which have not been well-characterised and could be misattributed to Aso-3. Our glass geochemical data from the caldera-forming eruptions from Aso indicate they produce compositionally heterogeneous deposits with a range of glass compositions. These ranges are distinctive and are a key defining feature, for each of the caldera-forming eruptions, including Aso-3. Further analysis of the Aso-2 caldera-forming event is required to strengthen future tephrochronological correlations during mid MIS 6. This work further highlights the importance of integrating geochemical fingerprinting, stratigraphic context, and independent age control when using Aso-3 as a time-stratigraphic marker.

## 6 Conclusions

This study establishes the stratigraphic position of the penultimate caldera-forming eruption of Aso and the utility of Aso-3 as a regional time-stratigraphic marker. New tephrostratigraphic and geochemical evidence from Lake Suigetsu (central Japan) and marine core U1427A (Sea of Japan) places the eruption in the terminal phase of MIS 6, with an estimated age of ∼133 ka. The heterogeneous glass shard compositions of Aso-3, mirrors that those observed in other caldera-forming events of Aso and help distinguish this tephra from other widespread eruptions dispersed during MIS 6 and 5. The alignment of the terrestrial and marine records shows that Aso-3 predates the MIS 5e high-stand and occurred just prior to regional climatic reorganisation of the deglacial. The Aso-3 tephra therefore offers a powerful isochron for linking diverse depositional environments and proxy systems across East Asia. Future identification offers significant opportunities to resolve the asynchronous pacing and structure of Termination II, particularly with respect to rates of environmental change and climate system feedbacks that are relevant for understanding aspects of future climate change.

## CRediT author statement

D. McLean: Conceptualization, Data Curation, Formal Analysis, Funding acquisition, Investigation, Project Administration, Methodology, Resources, Validation, Visualisation, Writing – orginal draft, Writing – review and editing. P. G. Albert: Conceptualization, Data Curation, Formal Analysis, Funding acquisition, Investigation, Project Administration, Methodology, Resources, Supervision, Validation, Visualisation, Writing – review and editing. G. Jones: Data Curation, Formal Analysis, Investigation, Methodology, Writing – review and editing. R. A. Staff: Data Curation, Formal Analysis, Investigation, Methodology, Writing – review and editing. A. Francke: Data Curation, Formal Analysis, Investigation, Visualisation, Writing – review and editing. S. O. Vineberg: Data Curation, Formal Analysis, Investigation. J. Tyler: Data Curation, Formal Analysis, Investigation, Writing – review and editing. M. Saito-Kato: Data Curation, Formal Analysis, Investigation, Methodology, Writing – review and editing. T. Sagawa: Data Curation, Formal Analysis, Investigation, Validation, Writing – review and editing. K. Kaneko: Data Curation, Formal Analysis, Investigation, Resources, Validation, Writing – review and editing. H. Buckland: Data Curation, Formal Analysis, Investigation. T. Suzuki: Investigation, Resources, Validation, Writing – review and editing. J-I. Kimura: Data Curation, Formal Analysis, Investigation, Resources, Writing – review and editing. Q. Chang: Data Curation, Formal Analysis, Investigation, Resources, Writing – review and editing. Y. Miyabuchi: Data Curation, Resources, Validation, Writing – review and editing. C. J. Manning: Resources, Writing – review and editing. K. Yamada: Data Curation, Formal Analysis, Investigation, Software, Validation, Writing – review and editing. I. Kitaba: Data Curation, Formal Analysis, Investigation, Validation, Writing – review and editing. K. Ikehara: Data Curation, Formal Analysis, Investigation, Validation, Writing – review and editing. T. Nakagawa: Data Curation, Formal Analysis, Funding acquisition, Investigation, Project Administration, Software, Validation, Writing – review and editing. V. C. Smith: Conceptualization, Data Curation, Formal Analysis, Funding acquisition, Investigation, Methodology, Project Administration, Resources, Supervision, Validation, Writing – review and editing.

## Declaration of competing interest

The authors declare that they have no known competing financial interests or personal relationships that could have appeared to influence the work reported in this paper.

## Data Availability

All data and/or code is contained within the submission.
